# Deciphering Candida’s Genomic Influence on Oral Squamous Cell Carcinoma: A Bioinformatics Approach

**DOI:** 10.31557/APJCP.2026.27.1.327

**Published:** 2026-01-22

**Authors:** Jothiha Shree, Suganya Panneer Selvam, Nitya Krishnasamy, Deepak Pandiar

**Affiliations:** *Department of Oral Pathology & Oral Biology, Saveetha Dental College and Hospital, Saveetha Institute of Medical and Technical Sciences, Saveetha University, India, Tamil Nadu, Chennai, India. *

**Keywords:** Bioinformatics, candida, cancer research, Th17 differentiation, chromosomal mapping, STRING database

## Abstract

**Background:**

Candida infection has been implicated in the progression of Oral Squamous Cell Carcinoma (OSCC). Understanding the molecular pathways and gene interactions involved in this process could provide new insights into the mechanisms underlying OSCC development and identify potential therapeutic targets. This study utilizes bioinformatics tools to analyze the genes associated with Candida infection and its role in the possible progression of the disease.

**Objective:**

To investigate the gene networks, enriched biological pathways, and chromosomal loci implicated in the progression of OSCC resulting from Candida infection using bioinformatics approaches.

**Methodology:**

Genes associated with OSCC and Candida infection were analyzed using the STRING database to identify protein-protein interaction (PPI) networks. Enrichment analysis of biological pathways was conducted, focusing on key immune and inflammatory processes. Gene ontology (GO) terms and functional annotations were examined. Chromosomal mapping of enriched genes was performed to identify significant genomic regions. Data visualization was achieved through static signaling plots and network diagrams, representing fold enrichment of targeted pathways and chromosomal positioning.

**Results:**

The cytokine-cytokine receptor interaction pathway exhibited the highest fold enrichment (~80-fold), indicating its significant role in immune modulation during OSCC progression. Other enriched pathways included viral protein interaction with cytokine receptors, cytosolic DNA-sensing pathways, and Th17 cell differentiation. Functional annotation revealed the involvement of macrophage proliferation, IL-33 signaling, and interferon responses, highlighting immune dysregulation in OSCC. Chromosomal mapping identified four enriched regions, primarily on chromosomes 2, 9, 12, and 16, indicating potential loci contributing to the disease’s molecular pathogenesis.

**Conclusion:**

The results suggest that immune signaling pathways, particularly cytokine-mediated interactions, play a critical role in Candida-mediated progression of OSCC. Chromosomal loci on chromosomes 2, 9, and 16 may harbor key genes involved in this process, potentially serving as targets for future therapeutic interventions. The findings further contribute to a deeper understanding of the immune-mediated mechanisms driving OSCC in the context of Candida infection.

## Introduction

Oral Squamous Cell Carcinoma (OSCC) represents the most common malignancy affecting the oral cavity, known for its aggressive nature and unfavorable prognosis as the disease advances. Despite extensive investigation of its risk factors, which include tobacco use, alcohol consumption, and human papillomavirus infection, the multifaceted etiology of OSCC remains incompletely understood [1]. Recent research has indicated a potential involvement of microbial infections, particularly fungal pathogens such as candida, in the onset and advancement of OSCC [2].

Candida albicans, a common commensal fungus in the oral microbiota, has been linked to various oral diseases, including candidiasis and OSCC [3]. Ongoing evidence suggests that persistent fungal infections and microbial dysbiosis may contribute to the carcinogenic process through chronic inflammation, immune modulation, and direct interaction with host tissues [4].

Advancements in bioinformatics and computational biology offer novel prospects for comprehending the intricate interactions between candida and OSCC. By harnessing high-throughput genomic and transcriptomic data, bioinformatics tools can unveil molecular signatures and interactions that may not be detected by conventional experimental methods [5-7]. This approach facilitates a comprehensive exploration of gene expression patterns, protein interactions, and signaling pathways associated with the role of candida in OSCC.

In this context, our study aims to apply bioinformatics techniques to investigate the involvement of candida in OSCC. Through comprehensive analysis of available genomic datasets and integration of protein-protein interaction networks, we seek to identify key candida-related genes and elucidate their potential impact on OSCC progression. Understanding these interactions could provide valuable insights into the role of microbial factors in oral cancer and pave the way for novel diagnostic and therapeutic strategies.

## Materials and Methods

### Data Collection

Gene Expression Data: Publicly available gene expression data for OSCC and candida infection were retrieved from relevant databases (NCBI reference sequence database). The Cancer Genome Atlas (TCGA) provided gene expression profiles for OSCC samples, while candida-related gene data were sourced from the candida Genome Database.

Database Access: The String database (STRING-db.org, version – 12.0) was utilized for protein-protein interaction (PPI) analysis. This database integrates known and predicted interactions for candida-related proteins and OSCC-associated genes.

### Bioinformatics Analysis

Protein-Protein Interaction Network Construction: The String database was employed to identify and map interaction networks. This involved data retrieval from the TCGA and Candida Genome Database, network construction, and visualization using Cytoscape (Cytoscape.org – version – 3.10.2). The visualization of these networks was performed using Cytoscape (version 3.10.2), which enabled a detailed view of the relationships between proteins and allowed further to explore the functional roles of specific proteins within the network. The analysis aimed to uncover potential hubs or key nodes that may contribute to OSCC progression in the context of candida infection.

### Enrichment Analysis

To explore the biological relevance of the identified proteins, enrichment analysis was performed, focusing on pathways and biological processes associated with Candida-related genes in OSCC. This included pathway enrichment analysis to identify which signaling pathways were most influenced by the presence of Candida. Fold enrichment calculations helped determine the magnitude of pathway involvement, while statistical significance was assessed using methods such as the false discovery rate (FDR). Data visualization techniques, including static signaling plots and interactive network diagrams, were used to present the findings clearly, making it easier to interpret the complex biological interactions between OSCC-associated and Candida-related genes. These visualizations highlighted how Candida infection may alter key signaling pathways like immune response modulation and inflammation in OSCC progression.

### Chromosomal Mapping

To identify the chromosomal regions most enriched in OSCC-associated genes, Cytoscape (v3.8.2) was used for network integration with chromosomal data. The following steps were performed: Gene symbols were mapped onto specific chromosomal positions using the Ensembl Genome Browser. Enriched chromosomal loci were detected based on the concentration of OSCC-related genes in specific regions. Loci with higher concentrations of OSCC-related genes were considered regions of interest, potentially pointing to genomic hotspots involved in OSCC pathogenesis.

### Pathway Involvement

**Figure 1:**
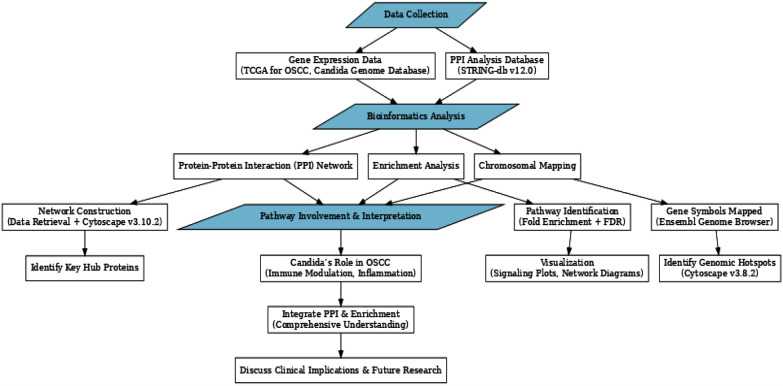
Flow Chart Showing the Methodology, Including Data Collection, Pathway Involvement, and Interpretation

The analysis focused on understanding the role of Candida in OSCC by examining how specific pathways, provided insights into the potential mechanisms linking Candida with OSCC progression. The combined insights from the interaction networks and pathway enrichment analyses were integrated to form a comprehensive understanding of Candida’s role in OSCC. The results were discussed in the context of existing research and potential implications for future studies and clinical applications. The complete methodology is visually represented in Figure 1 as a flowchart.

## Results

### Enrichment Pathways and Gene Interaction Network

**Figure 2:**
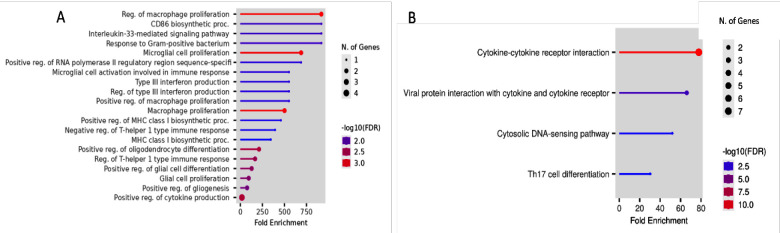
A Shows the STRING Database of the Genes Involved in Candida Progression to OSCC & B Shows the Fold Enrichment of Pathways Targeted for the Progression of Candida to OSCC

The analysis of network interactions using the STRING database has revealed a substantial enrichment of a set of genes in multiple biological pathways (Figure 2A). Notably, the cytokine-cytokine receptor interaction pathway exhibited the highest fold enrichment (~80-fold) and an FDR-adjusted p-value below 0.05. This suggests a substantial interaction between cytokines and their receptors, potentially contributing to immune modulation in the transition from Candida infection to OSCC. Furthermore, the enrichment of viral protein interaction with cytokines and cytokine receptors, cytosolic DNA-sensing pathways, and Th17 cell differentiation implies a viral or immune-mediated mechanism underlying OSCC progression (Figure 2B). 

### Functional Annotations and Pathway Enrichment

Functional annotations and pathway enrichment analyses identified several immune-related processes, including macrophage proliferation, IL-33 signaling, and interferon responses. The most enriched biological processes included positive regulation of macrophage proliferation, indicating a role of the innate immune system in the inflammatory microenvironment of OSCC. Additionally, enriched pathways such as MHC class I biosynthesis, oligodendrocyte differentiation, and cytokine production regulation underscore the complex immune interactions involved in tumor progression.

### Chromosomal Mapping of Enriched Genes

**Figure 3:**
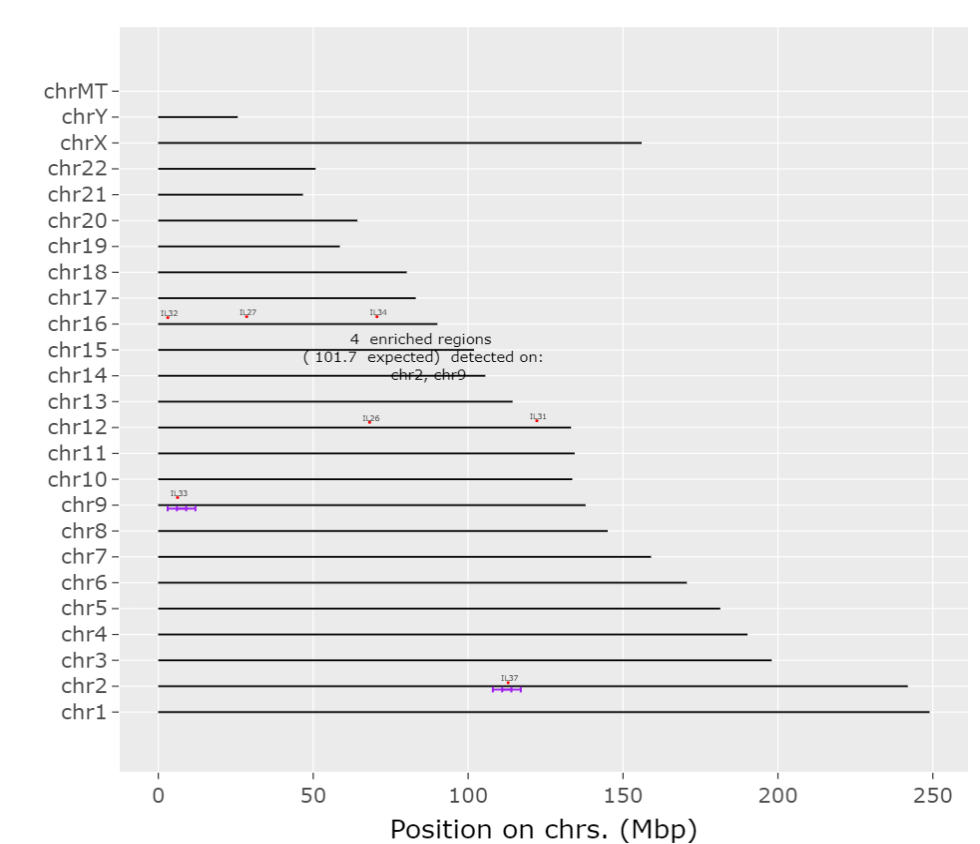
Shows Chromosomal Mapping of the Enriched Genes Involved in the Pathogenesis of OSCC by Candida

Chromosomal mapping of the differentially expressed genes revealed enriched regions primarily on chromosomes 2, 9, and 16 (Figure 3). These regions correspond to loci known to harbor genes involved in immune regulation and cancer progression. Notably, four specific regions showed significant enrichment, with the highest density found on chromosome 2 at approximately 100 Mbp. This integration of pathway enrichment and chromosomal localization indicates a strong association between immune signaling dysregulation and the onset of OSCC following Candida infection.

### Gene Interaction Networks

Several key genes involved in the immune response and inflammation appear central to the network, such as TLR2, TLR4, IL6, and CXCL8 (IL-8). These genes are often implicated in immune signaling pathways, especially about pathogen recognition and cytokine production, which are key processes in the body’s response to Candida infection. 

### Pathway Enrichment Analysis

**Figure 4:**
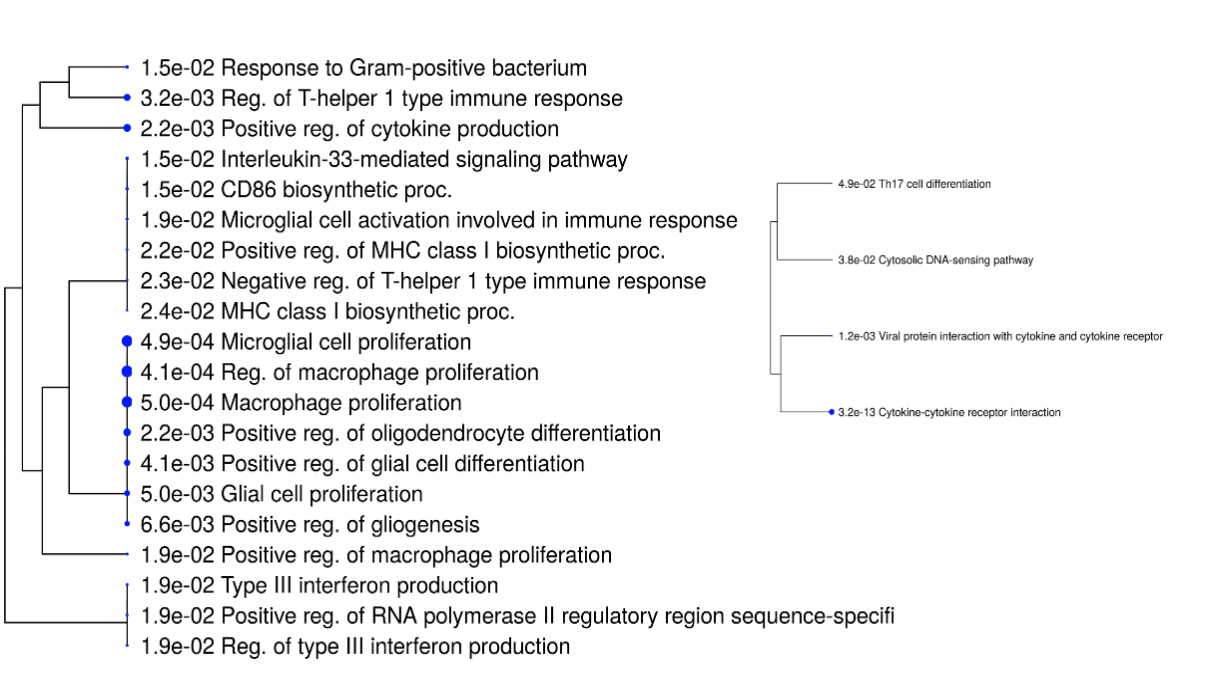
Shows Key Pathways Highlighting the Role of Candida in OSCC

The bioinformatics analysis revealed marked enrichment in several key pathways related to immune response and inflammation, highlighting the intricate role of Candida in OSCC (Figure 4).

### Th17 Cell Differentiation Pathway

The identification of genes including IL17A, IL17F, and IL22 suggests the activation of the Th17 cell pathway, aligning with the findings of the study, which implicated Th17-mediated inflammation in OSCC. This implies that candida may manipulate this response to create a pro-inflammatory environment conducive to carcinogenesis.

### Cytosolic DNA Sensing Pathway

**Figure 5:**
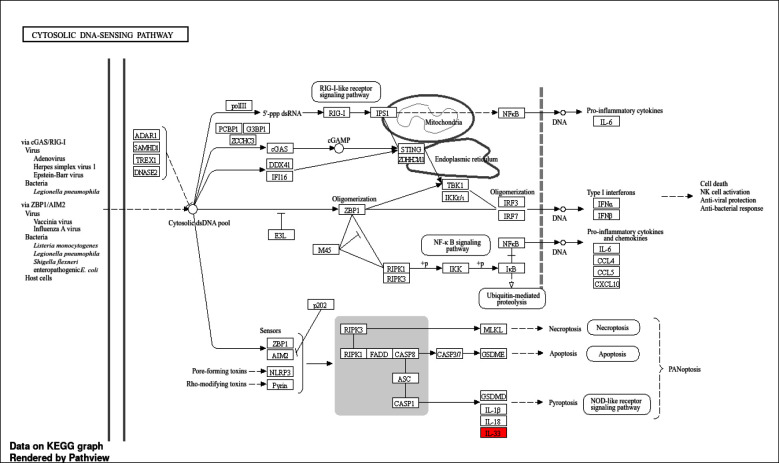
Shows the Influence of Candida in the Pathogenesis of the OSCC via Cytosolic DNA-Sensing Pathway

This analysis has also brought to light the significant enrichment of the cytosolic DNA sensing pathway. This pathway plays a crucial role in the innate immune response by identifying foreign DNA within the cytosol and activating immune responses (Figure 5). Enriching this pathway suggests that candida infection may stimulate innate immune mechanisms in OSCC, possibly by recognizing fungal DNA or associated molecular patterns. Cytokine-Cytokine Receptor Interaction Pathway 

**Figure 6:**
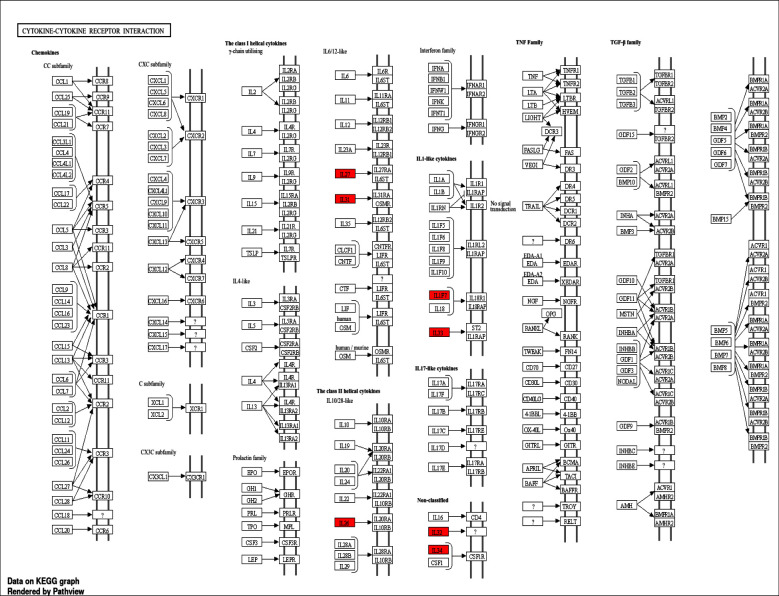
Shows the Cytokine-Cytokine Receptor Interaction Pathway Promoted by Candida in OSCC

This analysis underscores the significant involvement of the cytokine-cytokine receptor interaction pathway. Notably, genes such as IL6, CXCL8, IL10, IL17A, and CCL5 exhibit strong interconnections. These cytokines serve as critical mediators of inflammation, and their heightened expression may contribute to chronic inflammation, a pivotal factor in the progression of OSCC. Specifically, IL-6 is a well-established instigator of cancer-related inflammation (Figure 6). Cytokines and their corresponding receptors play vital roles in regulating immune responses and inflammation. The pronounced enrichment of this pathway indicates that Candida might impact OSCC progression by modulating cytokine signaling. 

### Viral Protein Interaction with Cytokine and Cytokine Receptor Pathway

The pathway associated with viral protein interactions with cytokines and cytokine receptors was found to be significantly enriched. Although typically linked to viral infections, the enrichment of this pathway in the context of Candida infection may indicate a cross-talk between fungal proteins and the host’s cytokine signaling mechanisms. This interaction could potentially intensify the inflammatory and immune responses, thereby contributing to the carcinogenic process in OSCC.

### Toll-Like Receptors (TLRs)

Genes like TLR2, TLR4, and TLR9 are central in the network, suggesting their critical role in recognizing fungal pathogens like Candida and initiating innate immune responses. TLR signaling can trigger the production of inflammatory cytokines that shape the tumor microenvironment. The ongoing production of inflammatory cytokines can foster a protumorigenic environment by attracting immune cells such as neutrophils and macrophages. Instead of eradicating cancer cells, these immune cells may facilitate tumor growth through the secretion of growth factors, matrix-degrading enzymes, and additional cytokines.

### Clustered gene groups

**Figure 7:**
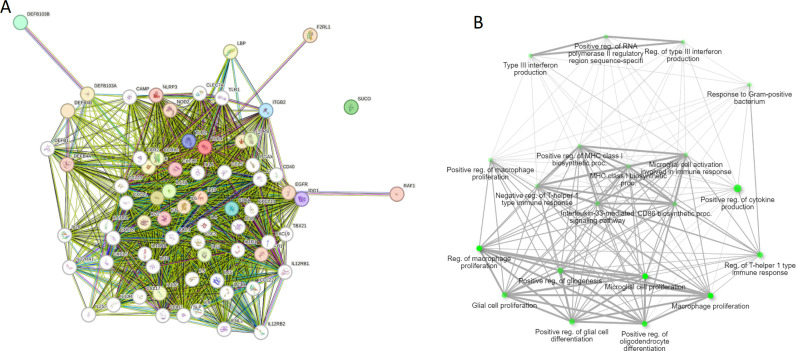
A Shows the STRING Database of the Genes Involved in Candida Progression to OSCC & 7B Shows the Network of Signaling Pathways

Certain genes showed IL1A, IL1B, and IL1RN (IL-1 receptor antagonist), are known to form close clusters and are integral to the interleukin signaling pathway. This pathway has been identified as a significant contributor to OSCC. Disruptions in the IL-1 pathway have the potential to induce heightened inflammation and advance cancer development. Additionally, DEFB103A and other defensins (e.g., DEFB1 and DEFB4A) were seen as somewhat peripheral but are linked to central nodes (Figure 7A). These genes encode antimicrobial peptides that can modulate immune responses and have been associated with mucosal immunity. While their relevance in OSCC, particularly in response to fungal infections, may be substantial, further research is necessary for comprehensive understanding. 

### Network Analysis

#### Comprehensive Signaling

A comprehensive view of the molecular interactions between Candida and OSCC was provided through the network analysis of signaling pathways (Figure 7B). The integration of pathways such as Th17 cell differentiation, cytosolic DNA sensing, cytokine-cytokine receptor interactions, and viral protein interactions with cytokines revealed a complex web of interactions. This network underscores the multifaceted role of Candida in modulating immune responses and inflammation in OSCC.

### Potential Therapeutic Targets

The network analysis revealed that EGFR and TLR4 are significant potential therapeutic targets. EGFR, known for its role as a therapeutic target in OSCC, shows prominent interaction with Candida infection, suggesting an area worthy of further investigation. Additionally, the presence of IDO1, a gene involved in tryptophan metabolism and immune tolerance, indicates a potential influence of Candida on immune evasion mechanisms in OSCC. This detailed analysis highlights several potential targets for therapeutic intervention, focusing on key nodes and interactions within enriched pathways. Novel strategies can be developed to target specific molecular mechanisms involved in Candida-associated OSCC, including cytokine signaling molecules, components of the cytosolic DNA sensing machinery, and regulators of Th17 cell differentiation.

### Static Signaling Plots and Network Diagrams

The static signaling plot depicted provides a simplified representation of an interconnected network of nodes, each representing a specific gene or protein involved in signaling pathways. The use of static signaling plots and network diagrams for data visualization effectively conveys the intricate interplay of the identified pathways. These visualizations offer valuable insights into the relative importance and interactions of different signaling components, facilitating the interpretation of Candida’s potential influence on OSCC progression. Genes such as DEF103B, RARA, and F2RL1 within the network appear more peripheral, indicating weaker or less direct interactions with the core inflammatory and immune pathways. Nonetheless, their connections suggest potential modulatory roles in specific contexts of infection or the immune response to Candida. The intricate interconnectivity implies that the pathway may be associated with a crucial biological process, potentially immune signaling or regulation of inflammation, particularly in the context of Candida infection and the progression of OSCC. The findings underscore the significance of cytokine signaling and immune response pathways in the context of Candida-associated OSCC. Understanding these interactions provides valuable insights into the mechanisms through which fungal infections may contribute to oral cancer. This understanding has implications for the development of targeted therapies and the enhancement of diagnostic strategies for OSCC with a microbial component. The results from this diagram suggest that the genes in this signaling network are likely central to the regulation of immune responses, especially in the context of Candida infections and their progression to OSCC. The presence of a highly connected node indicates a potential target for therapeutic intervention, where modulation of this gene’s activity could have a widespread impact on the downstream pathways, potentially altering the pro-inflammatory environment that promotes OSCC.

## Discussion

The findings from this study highlight the multifaceted role of Candida in the progression of OSCC, emphasizing its impact on immune response and inflammation. The bioinformatics analysis revealed significant enrichment in pathways related to immune modulation, particularly Th17 cell differentiation and cytokine signaling, suggesting that Candida may play a critical role in shaping the OSCC microenvironment.

The enrichment of the Th17 cell differentiation pathway in Candida-associated OSCC is particularly noteworthy. Th17 cells are known for their role in promoting chronic inflammation and tissue damage, which can facilitate cancer progression. Recent studies have documented that Th17 cells contribute to the development and progression of various cancers, including oral cancers, by secreting pro-inflammatory cytokines such as IL-17 [8,9]. Candida may influence OSCC progression by modulating Th17 responses, creating a pro-inflammatory environment that supports tumor growth and metastasis [10,11]. The significant enrichment of the cytosolic DNA sensing pathway suggests that Candida infection triggers an innate immune response through the detection of fungal DNA. This pathway, involving pattern recognition receptors such as STING (Stimulator of Interferon Genes), plays a critical role in the immune system’s ability to recognize and respond to microbial infections [12,13]. Activation of this pathway in the context of OSCC might contribute to an inflammatory response that, while initially protective, could inadvertently promote cancer development by sustaining chronic inflammation and immune activation. 

The prominent involvement of the cytokine-cytokine receptor interaction pathway underscores the importance of cytokine signaling in Candida-associated OSCC. Cytokines such as IL-6, IL-8, and TNF-α drive inflammation and have been implicated in the pathogenesis of various cancers, including OSCC [14,15]. The interaction between Candida-derived factors and host cytokine signaling pathways may exacerbate the inflammatory response, enhancing tumor cell proliferation and survival [16]. Although primarily associated with viral infections, the enrichment of pathways involving viral protein interactions with cytokines and their receptors in the context of Candida suggests a potential for cross-talk between fungal and host signaling mechanisms [17]. This cross-talk could further influence the inflammatory landscape and immune response in OSCC, highlighting the complex interplay between microbial factors and cancer biology [18].

The protein-protein interaction (PPI) network established within this investigation provides valuable insights into the molecular interplay among genes associated with Candida infection and OSCC. Our PPI network has identified several central genes, notably IL6, TNF, CXCL8, and CD44, which are recognized for their pivotal roles in both immune regulation and cancer development. These genes are heavily implicated in inflammation, cell proliferation, and migration, which are hallmark features of cancer. IL6 and TNF are pro-inflammatory cytokines that have been frequently associated with cancer-related inflammation [19]. IL6, in particular, stimulates the STAT3 signaling pathway, which is crucial for cancer cell survival, proliferation, and immune evasion [20]. High levels of IL-6 correlate with increased tumor size and advanced TNM stage, indicating a direct relationship with aggressive disease characteristics [21]. Overexpression of IL6 has been linked to unfavorable prognosis in OSCC patients, further underscoring its potential as a biomarker and therapeutic target. Similarly, TNF has been demonstrated to initiate a series of inflammatory signals that promote tumorigenesis. Its interaction with other inflammatory molecules in the PPI network underscores its central role in linking chronic infection with cancer progression [22]. CD44, another pivotal gene in the PPI network, is a well-established marker for cancer stem cells [23]. Its involvement in OSCC has been extensively documented, as it facilitates tumor cell migration, invasion, and metastasis. Given its role in maintaining cancer stem cells, CD44 could serve as a crucial therapeutic target to impede the recurrence and metastasis of OSCC in patients with Candida-associated infections.

The analysis of chromosomal mapping has identified a clustering of several genes associated with OSCC on specific chromosomes, notably chromosomes 2, 9, and 16. This finding is consistent with previous genomic investigations highlighting chromosomal abnormalities in these regions as characteristic of OSCC development [24]. For instance, frequent reports of loss of heterozygosity (LOH) and copy number variations (CNVs) on chromosome 9, particularly in genes involved in cell cycle regulation and apoptosis such as CDKN2A (p16), are common in OSCC patients. Additionally, chromosome 2 contains several oncogenes and tumor suppressor genes, including MYCN and ALPI, which have been implicated in various cancers, including OSCC. The co-localization of OSCC-associated genes on these specific chromosomal loci presents a novel avenue for comprehending the genetic determinants of OSCC in the context of Candida infection. Genes such as p16 and DAP-K show significant methylation in OSCC lesions, suggesting their involvement in cancer progression [25,26]. Further research focusing on chromosomal abnormalities in these regions has the potential to advance our understanding of the genetic predispositions that elevate the risk of developing OSCC subsequent to chronic Candida infection.

The insights gained from this study offer several potential avenues for therapeutic intervention. Targeting key pathways involved in Candida-associated OSCC, such as Th17 cell differentiation and cytokine signaling, could provide new strategies for managing this disease [27]. For instance, therapies aimed at modulating Th17 responses or inhibiting specific cytokines may help mitigate the inflammatory environment that supports tumor progression. Considering the role of Th17 cells in promoting inflammation and cancer progression, interventions targeting Th17 cell activity or cytokine production may yield significant benefits. Recent advancements in immunotherapy, such as monoclonal antibodies against IL-17 or IL-23, have exhibited potential in the treatment of autoimmune diseases and cancers associated with Th17 dysregulation [28,29]. These strategies could be further investigated in the context of OSCC to potentially disrupt the inflammatory microenvironment influenced by Candida. Targeting cytokine signaling pathways, specifically those involving IL-6 or TNF-α, may offer therapeutic advantages [30]. Studies have shown that inhibitors of these cytokines have been effective in treating various inflammatory diseases and cancers. These treatments have the potential to decrease the inflammatory response and enhance outcomes for OSCC patients with a microbial component [31]. Targeting CD44 could disrupt its interaction with hyaluronan, potentially reducing tumor cell motility and invasion [32,33]. The CD44/HAS1/MMP9 axis is crucial for ECM degradation and metastasis, suggesting that interventions aimed at this pathway may improve patient outcomes. While CD44 presents a compelling target for therapy, the complexity of its isoforms and interactions necessitates further research to fully understand its role in OSCC and associated infections.

Further investigation is required to corroborate these bioinformatics findings through empirical studies and clinical trials. It is imperative to delve into the precise mechanisms by which Candida affects Th17 cell responses and cytokine signaling in OSCC for the development of targeted therapies. Furthermore, examining the involvement of other microbial factors and their interactions with the tumor microenvironment could yield a more comprehensive understanding of the correlation between infections and cancer.

In conclusion, this research emphasizes the crucial role of Candida in influencing the OSCC microenvironment through immune modulation and inflammation. The identification of significant enrichment in pathways related to Th17 cell differentiation and cytokine signaling opens up new possibilities for developing innovative therapeutic approaches targeting these pathogenic interactions. Further exploration in this area holds the potential to enhance patient outcomes and create more effective treatments for OSCC. In summary, this study offers a comprehensive bioinformatics analysis of the molecular mechanisms driving the progression from Candida infection to OSCC. The discovery of key pathways and genes, including cytokine-cytokine receptor interactions, IL-6, TNF, and CD44, provides fresh insights into the inflammatory processes and genetic determinants of OSCC. These findings establish a solid foundation for future research focused on creating targeted therapies and preventive strategies for patients with Candida-associated OSCC.

## Author Contribution Statement

Jothiha Shree: Conceptualization, Data collection, and manuscript drafting. Suganya Panneer Selvam: Supervision, methodology validation, and critical revision of the manuscript. Nitya Krishnasamy: Data interpretation, visualization, and editing. Deepak Pandiar: Supervision, expert guidance, and final approval of the manuscript
