# Supplementary material for: Deciphering Candida’s Genomic Influence on Oral Squamous Cell Carcinoma: A Bioinformatics Approach
Source: Asian Pac J Cancer Prev. 2026 Jan 22;27(1):327–35. doi: 10.31557/APJCP.2026.27.1.327 (PMC13418028; doi:10.31557/APJCP.2026.27.1.327)
Supplement: Figure S1 [file APJCP-27-1-327-s001.pdf]

Deciphering Candida's Genomic Influence on Oral Squamous Cell Carcinoma: A Bioinformatics Approach

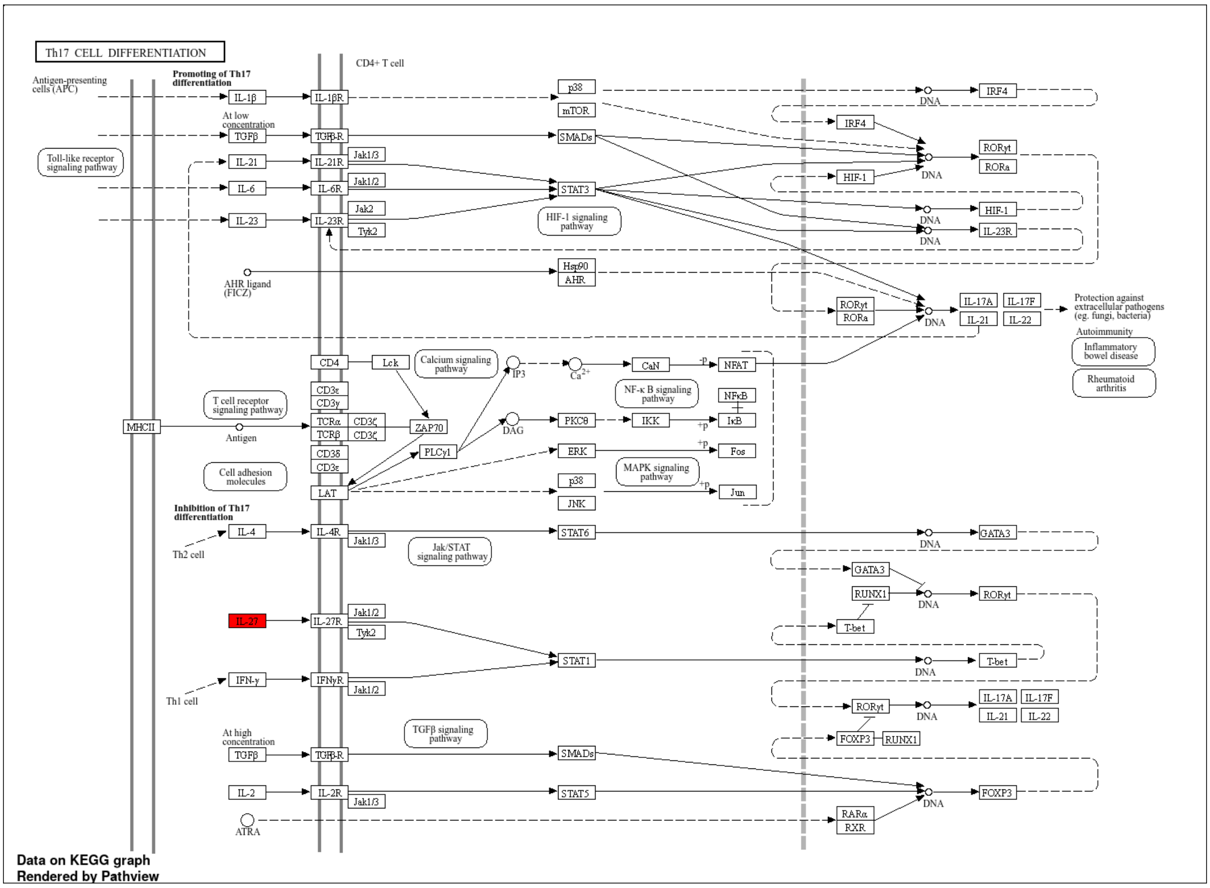

Th17 cell differentiation pathway promoted by candida in OSCC

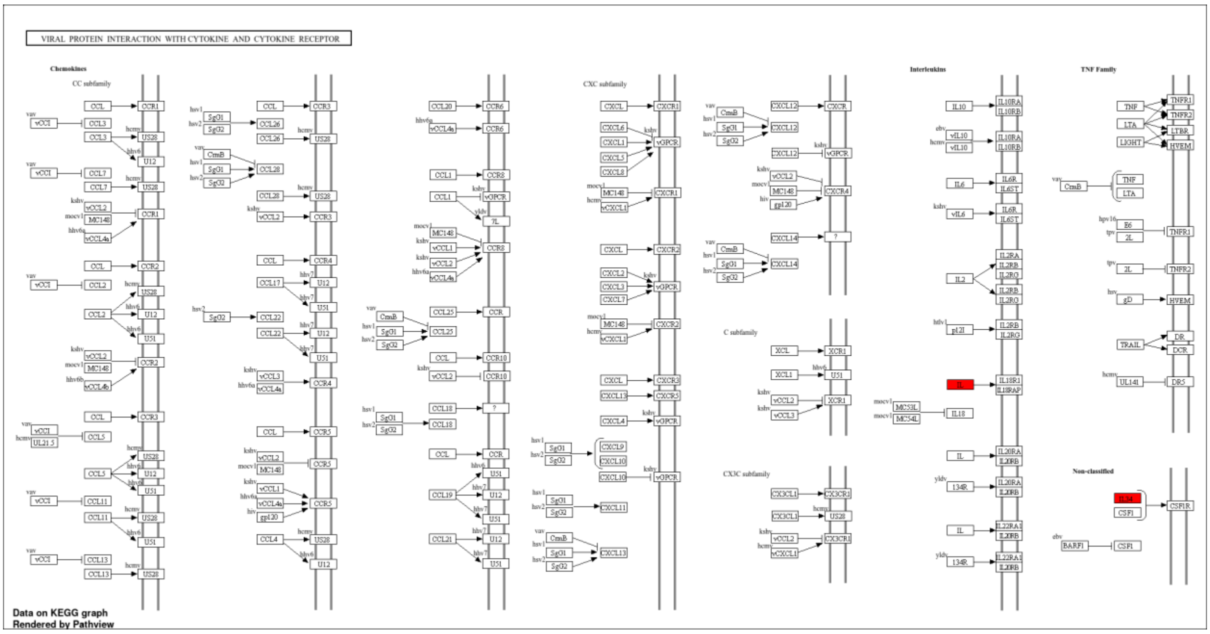

Viral protein interaction with cytokine and cytokine receptor

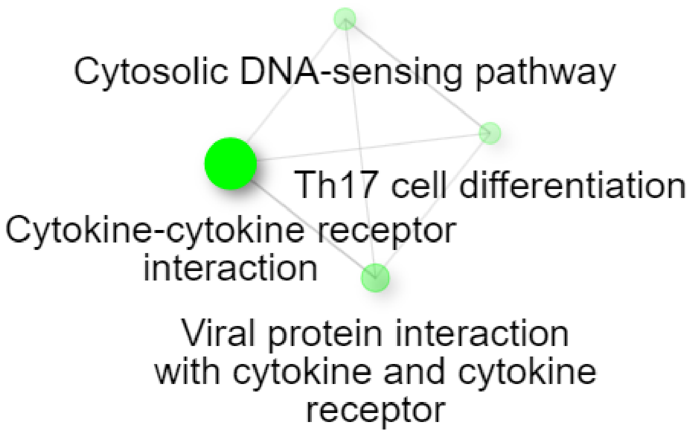

Static signaling plot showing the pathways influenced by Candida in progression of OSCC
